# Salvage gastrectomy for perforated gastric intramural metastasis after definitive chemoradiotherapy for distal esophageal squamous cell carcinoma: a case report

**DOI:** 10.1093/jscr/rjag932

**Published:** 2026-10-19

**Authors:** Jirat Leelapatanadit, Sawanya Sangthong, Rawat Waratchanont, Wichitra Asanprakit, Viriya Kaewkangsadan, Sukchai Satthaporn

**Affiliations:** Department of Surgery, Phramongkutklao Hospital, Thung Phaya Thai, Ratchathewi, Bangkok 10400, Thailand; Department of Surgery, Phramongkutklao Hospital, Thung Phaya Thai, Ratchathewi, Bangkok 10400, Thailand; Department of Surgery, Phramongkutklao Hospital, Thung Phaya Thai, Ratchathewi, Bangkok 10400, Thailand; Department of Surgery, Phramongkutklao Hospital, Thung Phaya Thai, Ratchathewi, Bangkok 10400, Thailand; Department of Surgery, Phramongkutklao Hospital, Thung Phaya Thai, Ratchathewi, Bangkok 10400, Thailand; Department of Surgery, Phramongkutklao Hospital, Thung Phaya Thai, Ratchathewi, Bangkok 10400, Thailand

**Keywords:** esophageal squamous cell carcinoma, gastric intramural metastasis, gastric perforation, chemoradiotherapy, salvage surgery

## Abstract

Gastric intramural metastasis from esophageal squamous cell carcinoma (ESCC) is rare, and chemotherapy-associated perforation is exceptionally uncommon. We report a case of a 67-year-old man with distal ESCC and a separate cardia squamous cell carcinoma (SCC). He declined esophagectomy and received definitive chemoradiotherapy (dCRT) with paclitaxel, carboplatin, and 50.4 Gy radiotherapy. During the second chemotherapy cycle, the cardia tumor perforated and was treated by emergency omental patch repair. Chemoradiotherapy was subsequently completed. Three months after completion of dCRT, the esophageal lesion showed a clinical complete response, whereas residual cardia disease persisted. Extended total gastrectomy with splenectomy achieved R0 resection; pathology showed a 6-cm pT3 moderately differentiated SCC with partial treatment response and 0/12 positive nodes. Postoperative nivolumab was administered. This case illustrates a staged salvage strategy for persistent gastric intramural disease after definitive chemoradiotherapy.

## Introduction

Intramural metastasis (IM) is an uncommon pattern of spread in esophageal squamous cell carcinoma (ESCC), probably through longitudinal submucosal lymphatics, and is associated with aggressive disease [1]. The Japanese Classification of Esophageal Cancer defines a gastric-wall metastasis clearly separated from the primary esophageal tumor as gastric IM (IM1-St) [2]. Perforation of gastric IM during anticancer therapy is exceptionally rare [3]. We describe the successful management of a perforated metastatic gastric tumor during definitive chemoradiotherapy (dCRT). This intervention enabled the patient to complete dCRT and subsequently undergo salvage gastrectomy for residual gastric disease.

## Case report

A 67-year-old man with unremarkable medical history presented with a 2-month history of progressive dysphagia and an 18-kg weight loss (from 78 to 60 kg). Esophagogastroduodenoscopy showed a circumferential distal esophageal mass at 34–37 cm from the incisors and a separate 5 × 5 cm ulcerative mass at the gastric cardia; the lesions were separated by approximately 5 cm and were not contiguous (Fig. 1). Biopsies from both lesions demonstrated moderately differentiated squamous cell carcinoma (SCC). Computed tomography (CT) showed asymmetrical circumferential lower-esophageal thickening up to 1.8 cm, paraesophageal lymph nodes up to 1.0 cm, a 5 × 5 cm cardia mass, and upper perigastric lymph nodes up to 1.0 cm, without distant metastasis. The esophageal lesion was clinically staged as cT3 with suspected regional nodal metastasis and no distant metastasis on conventional staging, with the separate gastric SCC considered gastric IM.

**Figure 1:**
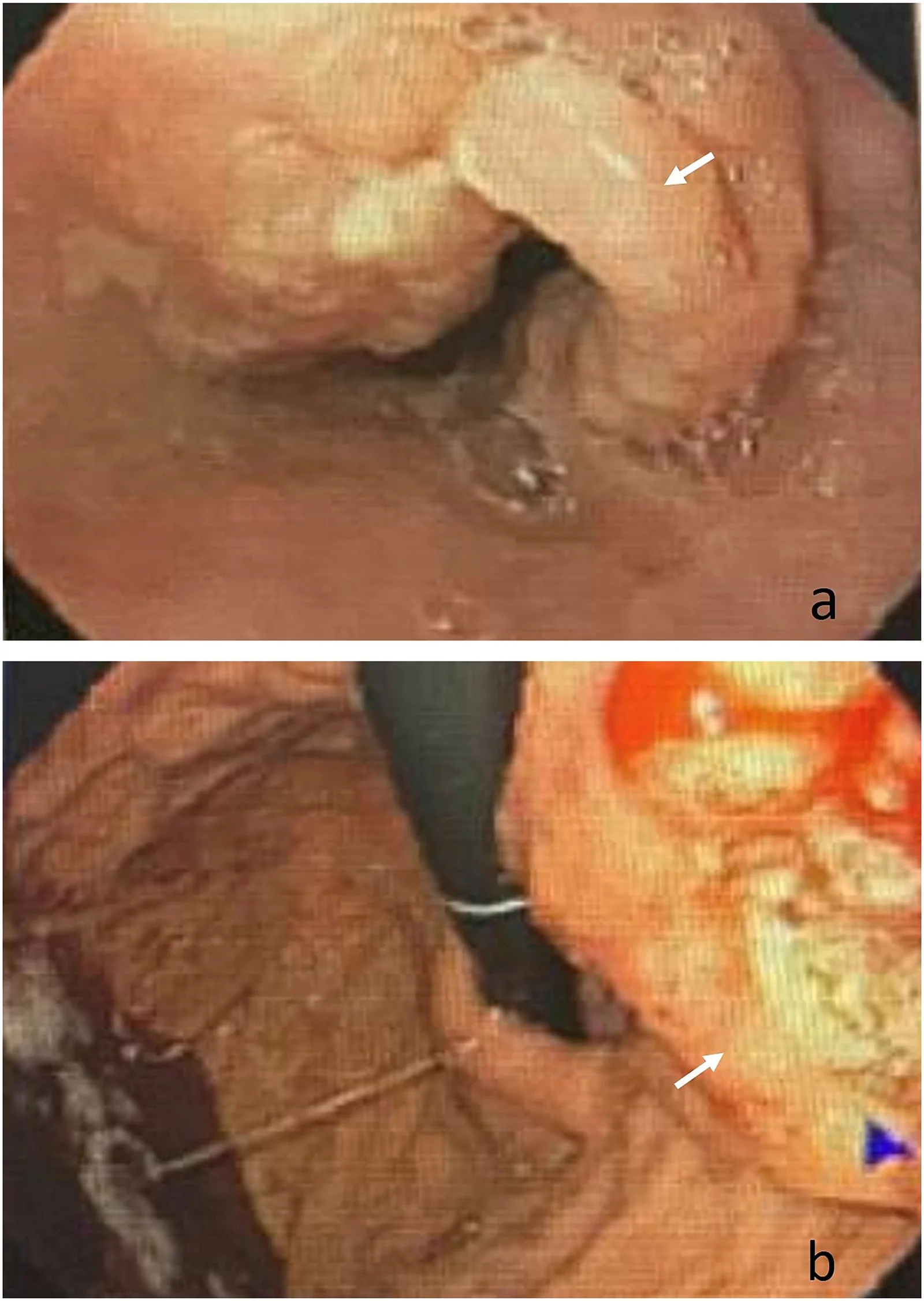
Esophagogastroduodenoscopy: (a) distal esophageal mass (arrow) and (b) mass at the gastric cardia (arrow).

Treatment options were discussed; however, the patient declined esophagectomy. Following multidisciplinary review, dCRT was initiated with concurrent paclitaxel/carboplatin and radiotherapy to a total dose of 50.4 Gy. During the second chemotherapy cycle, approximately 2 weeks after starting dCRT, the patient developed acute abdominal pain and generalized peritonitis. CT showed pneumoperitoneum with free air around the cardia mass (Fig. 2). Emergency exploratory laparotomy confirmed perforation of the cardia tumor, with a 2 × 2 cm defect. The defect was closed with an omental patch. After recovery, dCRT was resumed and completed.

**Figure 2:**
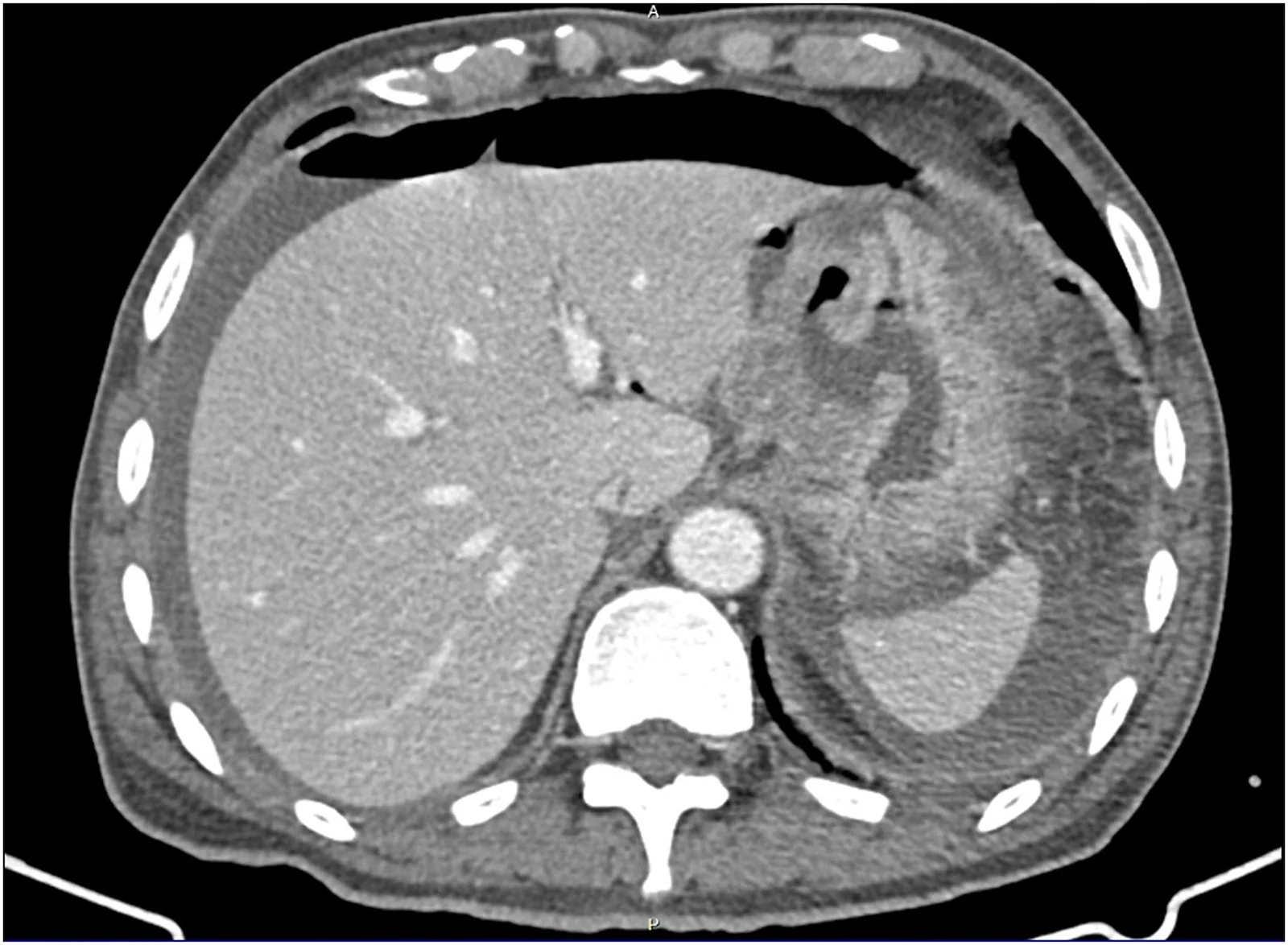
CT scan of the abdomen showing pneumoperitoneum with extraluminal free air adjacent to the gastric cardia mass.

Three months after dCRT, CT showed marked regression of esophageal thickening and subcentimeter mediastinal lymph nodes. The cardia mass decreased to 3 × 3 cm and perigastric lymph nodes became subcentimeter. Endoscopy showed a smooth esophageal scar stricture without residual mucosal tumor; random biopsies were negative. The esophageal lesion was considered a clinical complete response, whereas the cardia lesion remained a partial response (Fig. 3).

**Figure 3:**
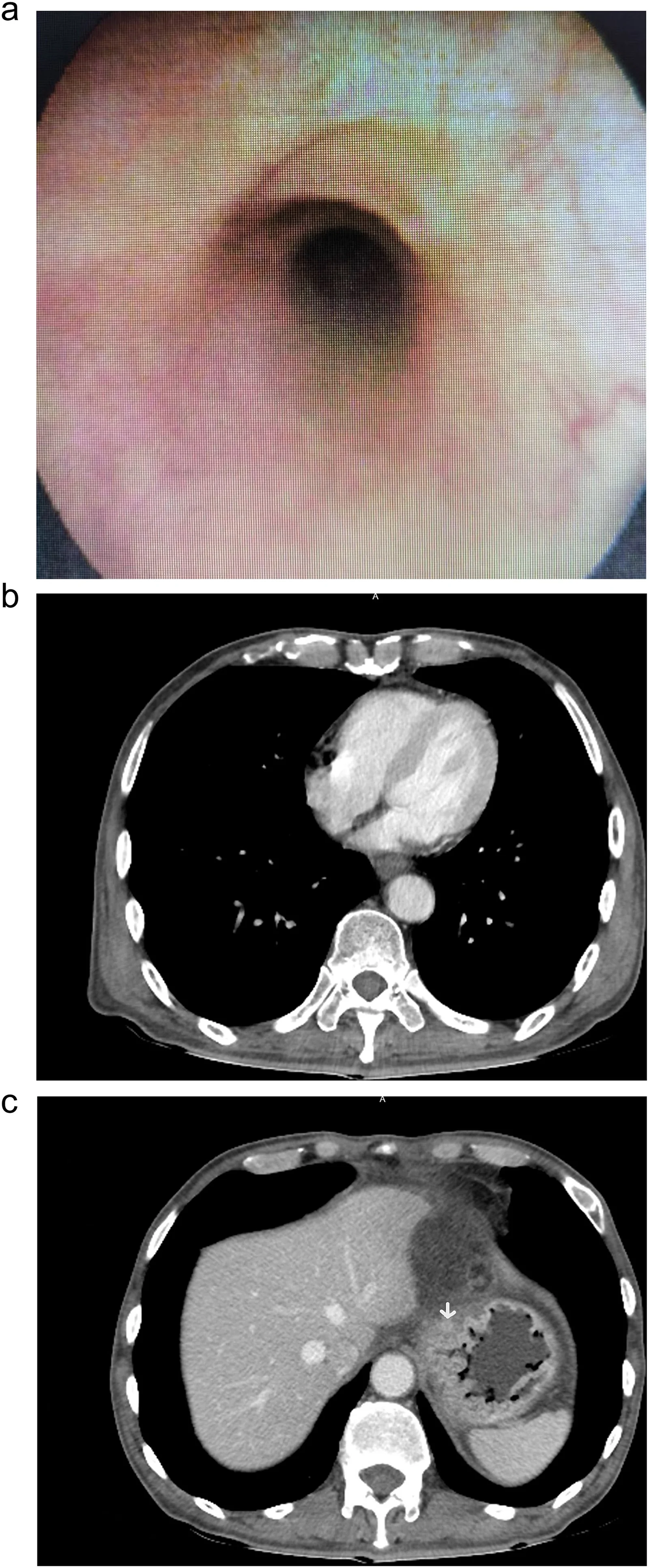
Restaging after definitive chemoradiotherapy (dCRT). (a) Esophagogastroduodenoscopy showing complete response of the esophageal lesion. (b) CT scan showing complete response of the esophageal lesion. (c) CT scan showing partial response of the gastric cardia lesion (arrow).

Salvage surgery was performed 3 months after dCRT. Severe adhesions and a cardia mass were encountered. Extended total gastrectomy with splenectomy and D1+ lymphadenectomy was performed. Histopathology demonstrated a 6-cm moderately differentiated SCC involving the cardia and fundus, with partial treatment response, negative margins, and 0/12 metastatic nodes (Fig. 4). Postoperatively, oral liquid intake was supplemented by enteral nutrition through the feeding jejunostomy, without any acute major complication. The patient subsequently received adjuvant nivolumab. At the 6-month follow-up after salvage surgery, the patient remained alive without evidence of local recurrence or distant metastasis.

**Figure 4:**
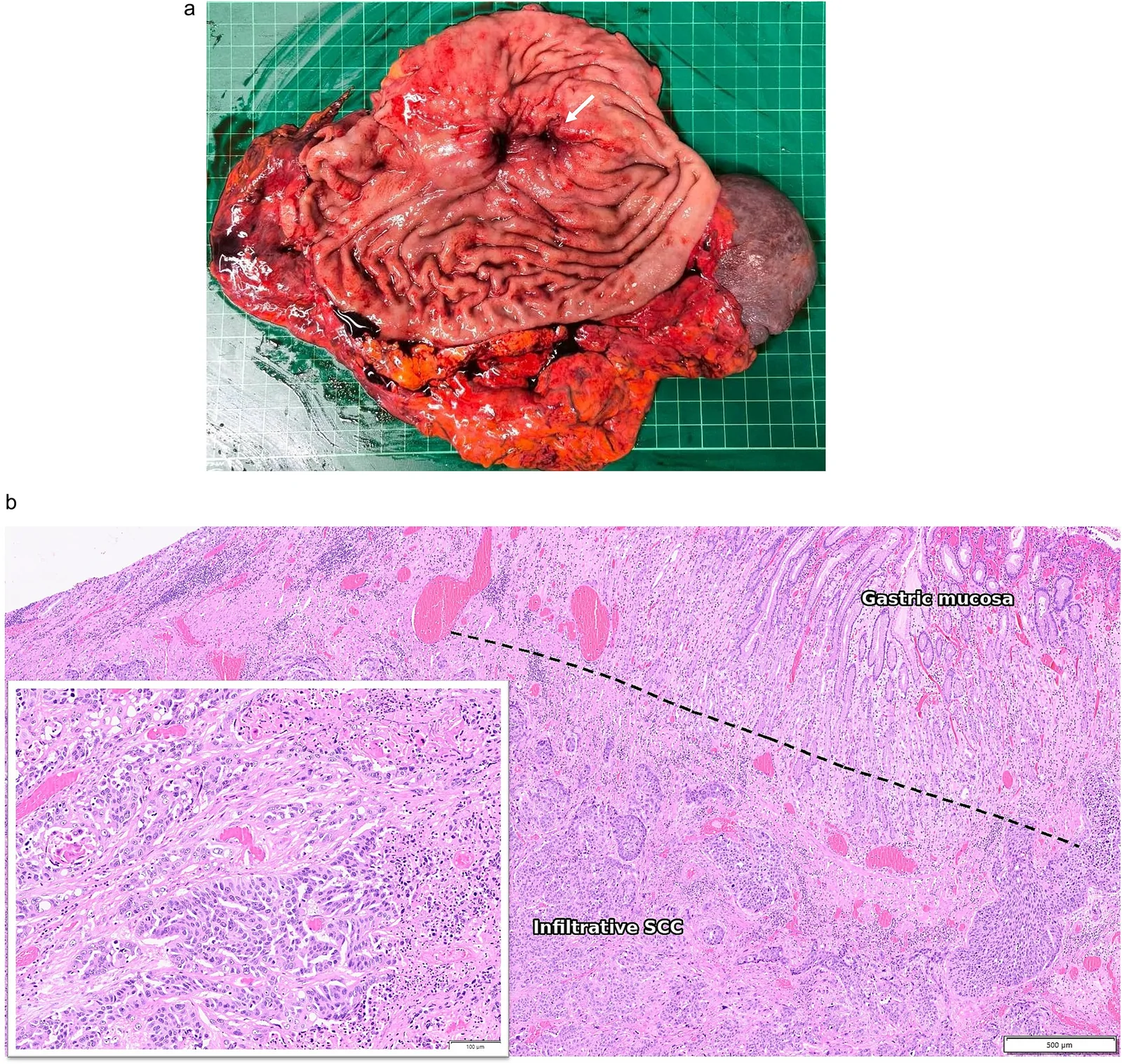
Gross surgical specimen following extended total gastrectomy with splenectomy. (a) Mucosal view of the opened stomach demonstrating the residual gastric cardia mass after definitive chemoradiotherapy. (b) Histopathological examination of the gastric cardia lesion showing infiltrative squamous cell carcinoma beneath relatively preserved gastric mucosa. The dashed line indicates the approximate interface between the gastric mucosa and the tumor-infiltrated gastric wall (H&E, ×20).

## Discussion

Gastric IM from ESCC is uncommon and must be distinguished from primary gastric SCC. In this patient, identical SCC histology, a cardia lesion clearly separated from the esophageal primary, and the known lymphatic route of intramural spread support gastric IM [1, 2]. Final confirmation is strongest when pathology demonstrates a gastric-wall/submucosal epicenter without an independent mucosal or intraepithelial SCC component.

Perforation during treatment is particularly rare. Okumura *et al*. reported gastric IM perforation during the second cycle of preoperative chemotherapy, followed by emergency omental repair and later radical resection [3]. Our case differs because the patient had declined esophagectomy, underwent dCRT, resumed treatment after emergency repair, achieved clinical complete response of the esophageal lesion, and subsequently underwent resection limited to persistent gastric disease.

Surgery after dCRT is technically demanding because radiation-related inflammation, fibrosis, and impaired tissue healing can increase postoperative morbidity. Most evidence concerns salvage esophagectomy; systematic reviews and surgical series nevertheless demonstrate that R0 salvage resection can provide meaningful survival in carefully selected patients, although anastomotic leakage and other complications remain important concerns [4, 5]. Reports of gastric resection after CRT for gastric-wall recurrence or intramural spread are limited but demonstrate technical feasibility [6]. In our patient, prior dCRT, perforation repair, and severe adhesions increased operative complexity, yet R0 extended total gastrectomy was achieved without an acute major complication.

CheckMate 577 established adjuvant nivolumab for residual pathological disease after neoadjuvant CRT followed by R0 resection of esophageal or gastroesophageal-junction cancer [7]. Our sequence—dCRT followed by salvage gastrectomy without esophagectomy—was not represented in that trial. Nivolumab has been reported to produce a marked response in residual/progressive gastric IM after surgery, supporting biological activity but not an adjuvant indication after R0 salvage resection [8]. Thus, postoperative nivolumab in this case represents extrapolation rather than an established standard. We found no report directly evaluating dCRT followed by R0 salvage gastrectomy and adjuvant nivolumab for gastric IM; accumulation of similar cases may help define the role of postoperative immunotherapy in this rare setting.

In conclusion, emergency control of perforation permitted completion of dCRT, followed by R0 salvage gastrectomy for persistent gastric IM. This staged approach may be feasible in highly selected patients without disseminated progression, but its oncologic value and the role of postoperative nivolumab require further study.

## References

[1] Ushimaru Y, Makino T, Tanaka K et al. Clinical significance of intramural metastasis as an independent prognostic factor in esophageal squamous cell carcinoma. Ann Surg Oncol 2023;30:5195–202. 10.1245/s10434-023-13464-w PMC10319648 37273025

[2] Mine S, Tanaka K, Kawachi H et al. Japanese classification of esophageal cancer, 12th edition: part I. Esophagus 2024;21:179–215. 10.1007/s10388-024-01054-y PMC11199297 38568243

[3] Okumura T, Shimada Y, Hojo S et al. Perforation of intramural gastric metastasis during preoperative chemotherapy in a patient with thoracic esophageal squamous cell carcinoma. Int J Surg Case Rep 2015;17:23–7. 10.1016/j.ijscr.2015.10.024 PMC4701806 26519812

[4] Yagi Y, Yoshida N, Baba Y et al. Salvage treatment after definitive chemoradiotherapy for esophageal squamous cell carcinoma. Ann Gastroenterol Surg 2021;5:436–45. 10.1002/ags3.12448 PMC8316734 34337292

[5] Markar SR, Jamel S. Salvage esophagectomy: safe therapeutic strategy? J Thorac Dis 2017;9:S799–808. 10.21037/jtd.2017.05.09 PMC5538991 28815077

[6] Kitano Y, Sugawara K, Miwa Y et al. Conversion surgery after chemoimmunotherapy for esophageal squamous cell carcinoma with gastric intramural metastasis: a case report and the literature review. J Surg Case Rep 2026;2026:rjag073. 10.1093/jscr/rjag073 PMC12911507 41710055

[7] Kelly RJ, Ajani JA, Kuzdzal J et al. Adjuvant nivolumab in resected esophageal or gastroesophageal junction cancer. N Engl J Med 2021;384:1191–203. 10.1056/NEJMoa2032125 33789008

[8] Wakasugi A, Kimura Y, Natsugoe K et al. Combined treatment with surgery and immune checkpoint inhibitor extended survival in a case of gastric intramural metastasis from esophageal cancer: a case report. Surg Case Rep 2023;9:118. 10.1186/s40792-023-01703-x PMC10293484 37358687

